# Environmental Factors Driving the Seasonal Dynamics of *Ixodes ricinus* and *Dermacentor reticulatus* in Eastern Poland

**DOI:** 10.3390/insects16050490

**Published:** 2025-05-02

**Authors:** Aneta Woźniak, Zbigniew Zając, Joanna Kulisz

**Affiliations:** Department of Biology and Parasitology, Medical University of Lublin, Radziwiłłowska 11, 20-080 Lublin, Poland

**Keywords:** *D. reticulatus*, *I. ricinus*, seasonal activity of ticks, tick-borne diseases, tick habitat

## Abstract

*I. ricinus* and *D. reticulatus* are among the most important vectors of pathogens causing tick-borne diseases in humans and animals. This study investigated their seasonal activity in Eastern Poland, a region with one of the highest incidence rates of tick-borne diseases nationwide. Additionally, we examined tick habitat preferences and the impact of abiotic factors on their activity patterns. To this end, we conducted systematic monitoring of tick activity between 2017 and 2019 in two ecologically distinct habitats: a mixed forest and a meadow. Using Generalized Additive Models (GAMs) and Generalized Linear Mixed Models (GLMMs), we analyzed the collected data. Our findings indicate that *I. ricinus* reaches peak activity in late spring, within a temperature range of 9.5–16.5 °C and relative humidity between 45.3% and 84.5%. In contrast, *D. reticulatus* demonstrated a broader thermal tolerance, remaining active at temperatures ranging from 1.0 °C to 32.6 °C and relative air humidity of 33.8–89.0%. Both temperature and humidity were found to be significant factors influencing tick activity. Moreover, *I. ricinus* and *D. reticulatus* exhibited distinct seasonal activity patterns throughout the year.

## Simple Summary

*Ixodes ricinus* and *Dermacentor reticulatus* are among the most epidemiologically significant tick species in Poland. In this study, we examined their habitat preferences and monitored their seasonal activity in response to air temperature and humidity. Our findings confirmed that both species can co-occur in forest and meadow habitats. We also demonstrated that *D. reticulatus* has a broader tolerance to temperature variations. Additionally, we confirmed that tick activity varies significantly throughout the year.

## 1. Introduction

Ticks (Ixodida) are arachnids of major epidemiological, veterinary, and medical importance, serving as one of the most significant vectors of pathogens that affect both humans and animals worldwide [1]. In Europe—and particularly in Poland—two ixodid species, *I. ricinus* and *D. reticulatus*, predominate in both abundance and epidemiological relevance [2]. These species transmit a wide spectrum of pathogenic microorganisms, including *Borrelia burgdorferi* sensu lato spirochetes (the agents of Lyme borreliosis), *Anaplasma phagocytophilum* (causing anaplasmosis), various *Babesia* spp. protozoa (responsible for babesiosis), agents of the spotted fever rickettsia group (e.g., *Rickettsia raoultii, R. helvetica*), *Francisella tularensis* (the cause of tularemia), and the tick-borne encephalitis virus (TBEV) [1,3,4,5,6,7,8,9].

In Poland, *I. ricinus* primarily inhabits forested areas, where high humidity and dense vegetation provide optimal conditions for the survival of all developmental stages [10,11]. In contrast, *D. reticulatus*, commonly known as the meadow tick, prefers open landscapes such as meadows, river valleys, and pastures. This species exhibits greater resistance to periodic desiccation and frequently co-occurs with *I. ricinus* in overlapping habitats, allowing them to parasitize the same hosts [12,13].

Previously conducted studies have suggested that the seasonal activity of ixodid ticks is influenced by both internal and external factors. Internal factors include hormonal regulation that directs tick development and life cycles, such as juvenile hormone and ecdysone, which influence both growth and behavioral adaptations related to host-seeking activity. External factors, including abiotic conditions such as temperature, humidity, photoperiod, and habitat type, may also play a crucial role in tick activity throughout the year [11,14,15].

The present study provides a comparative analysis of the activity patterns of *I. ricinus* and *D. reticulatus* across various habitats in Eastern Poland—a region characterized by one of the highest densities of these ticks in the country, a high prevalence of tick-borne pathogens, and a corresponding rise in the incidence of tick-borne diseases in humans [11,16,17]. The primary objective was to assess how abiotic factors, particularly temperature and relative air humidity, affect the seasonal activity of the studied species. Furthermore, we hypothesized that adult *I. ricinus* and *D. reticulatus* exhibit distinct activity rhythms depending on the type of habitat they occupy.

## 2. Materials and Methods

### 2.1. Study Area

**Figure 1:**
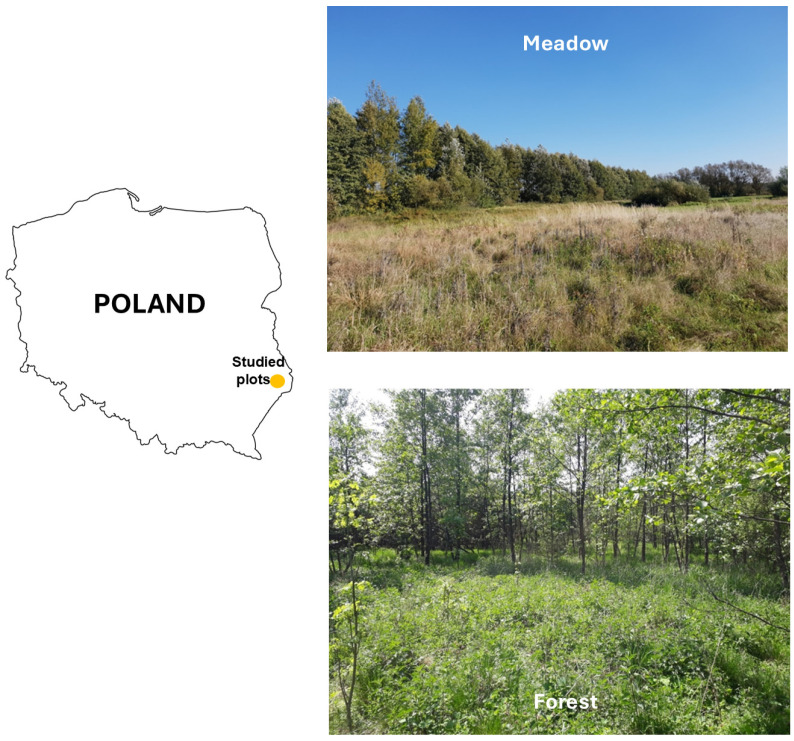
Study location and sampling sites. The research was conducted in Eastern Poland, where tick collections were carried out in two distinct habitat types: meadow (**top**) and forest (**bottom**). The meadow site is characterized by open grassland with scattered shrubs, while the forest site consists of a mixed deciduous habitat. The studied plots are marked on the map. Photo by Aneta Woźniak.

Field research was conducted in Eastern Poland (Figure 1). Tick collection sites were designated within the Eastern Roztocze mesoregion, which lies in a transitional temperate climate zone with distinct continental influences. This region is characterized by high variability in weather conditions due to its location at the intersection of oceanic influences from the Atlantic and continental influences from Eastern Europe [18].

The average annual air temperature in Eastern Roztocze ranges from approximately 7.0 to 7.5 °C, which is lower than in the neighboring lowland regions. The warmest month is July, with an average temperature of 18.0–19.0 °C, while the coldest is January, with an average temperature of −3.5 to −4.5 °C. Annual precipitation totals between 600.0 and 750.0 mm, making it one of the wettest areas in Eastern Poland. The highest precipitation occurs during the summer months (June–August), whereas the lowest is recorded in winter, particularly in February. Snow cover persists for an average of 70–80 days per year, and in higher elevations, it can last over 90 days. The growing season spans 205–215 days [18].

The region is predominantly forested, covering 65.5% of the area, with subcontinental mixed forests (*Tilio-Carpinetum*) as the dominant vegetation type. On the Eastern slopes, the Volhynian variant prevails, while on the western slopes, the Lesser Poland variant is present. In river valleys, Central European alder swamp forests (*Alnus glutinosa* habitats) are common. In the southern part of the region, beech forests with a mixture of fir and pine dominate, while eutrophic meadows occur in valley areas [18].

### 2.2. Tick Surveillance

Field research was conducted during the seasonal activity period of *D. reticulatus* and *I. ricinus* in Eastern Poland between 2017 and 2019. Tick collections were carried out biweekly, under favorable weather conditions—on days without precipitation (rain or snow) or strong winds. Each sampling session lasted one hour, during which ticks were collected from vegetation using the flagging method [2]. Additionally, during each collection, real-time weather conditions, including temperature and relative humidity, were measured using a DataLogger R6030 (Reed Instruments, Wilmington, NC, USA)).

In the laboratory, collected specimens were identified regarding species and developmental stages using a tick identification key [2]. This study focused exclusively on the activity of adult ticks from both species, while *I. ricinus* nymphs and larvae were excluded from the analysis.

#### Tick Collection Sites

The study sites were established within two ecologically distinct habitat types: a meadow and a mixed forest (Figure 1).

The meadow habitat was surrounded on three sides by mixed forest. One section of the area featured a natural depression, which facilitated periodic water accumulation. The dominant vegetation consisted of grasses (*Poaceae*) and sedges (*Cyperaceae*). The areas adjacent to the forest exhibited ongoing ecological succession, as evidenced by the presence of small shrubs and naturally regenerating birch saplings (*Betula* sp.) (Figure 1). During field research, proof of animal activity—potential tick hosts—was observed, including footprints, droppings, and fur.

The forest site was located within a woodland clearing, bordered on one side by a tree nursery where spruce trees were cultivated. The dominant vegetation included nitrophilous herbaceous species such as thistles (*Carduus* sp.) and plants from the nettle family (*Urticaceae*), the carrot family (*Apiaceae*), and the aster family (*Asteraceae*). The area was also covered by small shrubs and an understory composed of both coniferous and deciduous tree species (Figure 1). In terrain depressions, water periodically accumulated, particularly after heavy rainfall.

### 2.3. Statistical Analysis

In this study, we used a Generalized Additive Model (GAM) with the *mgcv* package to evaluate the effects of temperature, humidity, and seasonality on the activity of *I. ricinus* and *D. reticulatus*. Additionally, a Generalized Linear Mixed Model (GLMM) with the *lme4* package was applied to assess the impact of habitat type (meadow vs. forest) on the activity of the studied tick species. All analyses were conducted using R software (R Core Team, 2024) [19,20].

#### 2.3.1. Generalized Additive Model (GAM) Analysis of Tick Activity

GAM was used to assess the effects of temperature, humidity, and seasonality on the activity of *I. ricinus* and *D. reticulatus*. The dataset was derived from field collections conducted in both meadow and forest habitats, with tick counts recorded separately for males and females of each species. Environmental variables, including air temperature and humidity, were measured at the time of collection, while seasonality was classified based on the date.

Prior to modeling, the data underwent preprocessing to ensure accuracy and consistency. Temperature and humidity were treated as continuous variables, whereas seasonality was represented as a categorical variable corresponding to the month of collection. Tick counts for each species and sex served as the dependent variable in the analysis.

GAM was implemented to capture potential nonlinear relationships between tick activity and predictor variables. Tick count data were modeled using a Poisson distribution, with a negative binomial adjustment applied when necessary to account for overdispersion. Model robustness was assessed through residual plots, which were examined for heteroscedasticity and autocorrelation. Partial dependence plots were generated to visualize the nonlinear effects of temperature, humidity, and seasonality, while sensitivity analyses were performed by systematically excluding individual variables to evaluate their independent contributions.

#### 2.3.2. Generalized Linear Mixed Model (GLMM) Analysis of Tick Activity in Relation to Habitat Type

GLMM was employed to evaluate the influence of habitat type (meadow vs. forest) on the activity of the collected tick species. Data were obtained from two sampling locations, with tick counts recorded separately for males and females of each species. The primary predictor variables included habitat type (categorical: meadow or forest), as well as air temperature and humidity (continuous). Tick counts were initially modeled using a Poisson distribution, and in cases of overdispersion (variance exceeding the mean), a Negative Binomial GLMM was applied to improve model fit.

Model residuals were examined to assess the adequacy of the fit, ensuring that assumptions were met. If the estimated coefficient for habitat type significantly differed from zero, it was considered a significant predictor of tick activity.

## 3. Results

### 3.1. Occurrence and Abundance of Ticks

**Figure 2:**
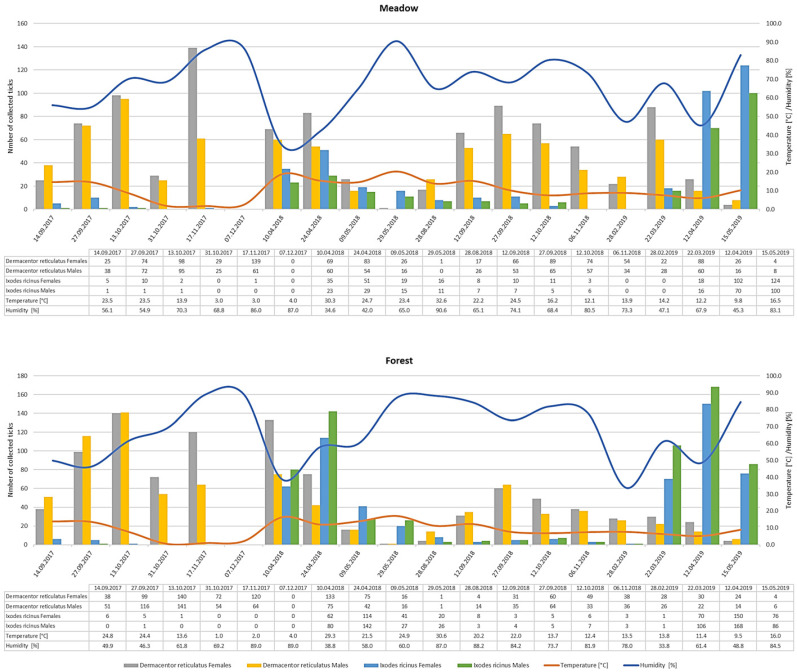
Seasonal abundance and activity of *I. ricinus* and *D. reticulatus* ticks. The figure presents the temporal dynamics of tick activity throughout the studied period of 2017–2019.

A total of 5459 adult ticks were collected during the study period, comprising 3522 *D. reticulatus* and 1937 *I. ricinus* individuals. *D. reticulatus* exhibited activity across a broad temperature range (1.0–32.6 °C), with peak abundance observed between 12.0 °C and 25.0 °C. In contrast, *I. ricinus* was more active at lower temperatures, reaching its highest densities between 9.5 °C and 16.5 °C (Figure 2).

Similarly, air humidity significantly influenced tick activity. *I. ricinus* preferred more humid conditions, with activity levels highest within a range of 45.3–84.5% relative air humidity, whereas *D. reticulatus* demonstrated a broader tolerance, remaining active in humidity levels ranging from 34.6% to 90.6% (Figure 2).

*D. reticulatus* was the dominant species in both meadow and forest habitats, reaching peak abundances of up to 200 individuals in the meadow and 281 in the forest during a single collection event. However, *I. ricinus* was more consistently present in the forest, where it displayed greater population densities, particularly during spring (Figure 2).

Both species exhibited distinct seasonal fluctuations in abundance. *D. reticulatus* followed a bimodal activity pattern, with peak occurrences in early spring (March–April) and autumn (September–November). In contrast, *I. ricinus* was primarily active in spring, with the highest numbers recorded in April and May. During the winter months, both species showed a marked decline in activity, with no specimens collected in December (Figure 2).

### 3.2. Impact of Environmental Factors on Tick Activity

#### 3.2.1. Impact of Air Temperature on Tick Activity

**Figure 3:**
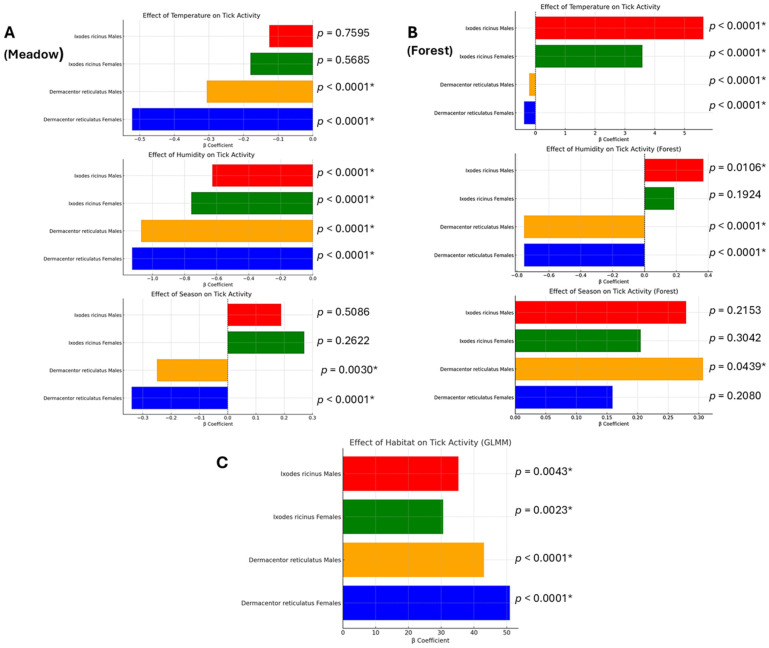
Effects of environmental factors and habitat type on the activity of *I. ricinus* and *D. reticulatus*. (**A**,**B**) The results of Generalized Additive Models (GAMs) evaluating the influence of air temperature, humidity, and seasonality on tick activity in the meadow (**A**) and forest (**B**) habitats. (**C**) The results of a Generalized Linear Mixed Model (GLMM) assessing the impact of habitat type on tick activity. Asterisks indicate statistically significant effects (*p* < 0.05).

The effect of temperature on tick activity varied across species, sex, and habitat. In the meadow, *D. reticulatus* males and females exhibited a statistically significant negative response to increasing temperatures (*p* < 0.0001), indicating a decline in activity at higher temperatures. A similar trend was observed in *I. ricinus*, although the effect was not statistically significant. Conversely, in the forest habitat, temperature had a significant impact on all groups (*p* < 0.0001), with *I. ricinus* displaying a notably stronger positive response to increasing temperatures (Figure 3).

#### 3.2.2. Impact of Relative Air Humidity on Tick Activity

Humidity significantly influenced the activity of both tick species across habitats. In the meadow, all groups showed a strong negative correlation with humidity (*p* < 0.0001), indicating decreased activity under higher moisture conditions. A similar pattern was observed for *D. reticulatus* in the forest, where humidity had a significant negative effect (*p* < 0.0001). In contrast, *I. ricinus* females in the forest did not exhibit a statistically significant relationship with humidity (*p* = 0.1924), suggesting that their activity may be regulated by other environmental factors (Figure 3).

#### 3.2.3. Impact of Season on Tick Activity

The influence of seasonality on tick activity was more pronounced in *D. reticulatus* than in *I. ricinus*. In the meadow, *D. reticulatus* males (*p* = 0.0030) and females (*p* < 0.0001) were significantly affected by seasonal changes, whereas *I. ricinus* showed no significant variation in activity across seasons. In the forest habitat, only *D. reticulatus* males exhibited a significant response to seasonality (*p* = 0.0439), while no such effect was detected in other groups (Figure 3).

#### 3.2.4. Impact of Habitat Type on Tick Occurrence

The GLMM analysis demonstrated a significant effect of habitat type on tick occurrence across all groups. *I. ricinus* males (*p* = 0.0043) and females (*p* = 0.0023) showed a strong habitat preference, with the forest being the more favorable environment. Similarly, *D. reticulatus* males and females also exhibited a strong habitat effect (*p* < 0.0001), with higher activity in the meadow (Figure 3).

## 4. Discussion

In this study, we analyzed the impact of environmental factors on the activity of *I. ricinus* and *D. reticulatus* in relation to the ecological habitat type. The obtained data indicate significant differences between these species in terms of habitat preferences, seasonal activity patterns, and responses to temperature and humidity (Figure 2 and Figure 3).

The results of this study align with our previous long-term monitoring of *D. reticulatus* in Eastern Poland, confirming the species’ strong habitat preferences and widespread presence in the region [21,22,23]. We found that *D. reticulatus* forms a dense population, particularly favoring unmanaged meadows undergoing ecological succession—an observation consistent with previously published reports [1,24,25].

Notably, while *D. reticulatus* was abundant in the meadow habitat, we also recorded a substantial population in the forest (Figure 2). However, GLMM analysis revealed that this habitat type is primarily preferred by *I. ricinus* (Figure 3), which is in line with previous findings indicating the species’ affinity for humid forest environments [26]. In contrast, adult *I. ricinus* did not exhibit increased activity in the meadow during periods of the highest relative air humidity throughout the year (Figure 2 and Figure 3). We attribute this to the extended monitoring period, which went beyond the commonly recognized seasonal activity window for this species in Poland [11]. Our fieldwork included November–December and March, months characterized by the lowest temperatures recorded during the study, accompanied by high relative air humidity levels (Figure 2).

We are aware that the limitation of this study is the lack of continuous, year-round sampling, which resulted from weather-related constraints. Tick collections were not carried out during periods of snow cover and frost (January–March/April) or during extreme summer conditions like high temperatures and drought. These conditions significantly reduce tick activity and could have biased the results if sampling had been conducted.

While this limits the completeness of the seasonal dataset, we prioritized biologically relevant data collection. Additionally, seasonal inactivity—such as summer diapause in *D. reticulatus* and reduced activity of *I. ricinus*—is well documented in this region [11,16,21,22,23]. Despite these constraints, our findings still offer valuable insight into tick seasonality under typical field conditions.

Notable differences in tick abundance were observed between the two habitats. The forest supported a higher overall density of *I. ricinus*, particularly in the spring, which suggests that this species prefers more humid, shaded environments. *D. reticulatus*, while dominant in both habitats, exhibited higher numbers in the meadow during autumn, indicating a possible preference for more open environments with fluctuating microclimatic conditions (Figure 2 and Figure 3).

Our study also confirmed the co-occurrence of *I. ricinus* and *D. reticulatus* in both meadow and forest habitats (Figure 2), which has significant implications for the epidemiology of tick-borne diseases. Given their overlapping host range, the probability of both species feeding on the same host increases, which may enhance the risk of pathogen transmission through co-feeding [16,27,28].

During our field studies, we observed that adult *D. reticulatus* remained active across a broad temperature range (1.0 –32.6 °C), with peak abundance recorded between 12 °C and 25 °C (Figure 2). In contrast, adult *I. ricinus* exhibited peak activity within a narrower range of 9.5–16.5 °C (Figure 2). In the forest habitat, temperature had a significant effect on all groups (*p* < 0.0001), with *I. ricinus* showing a stronger positive response to temperature fluctuations than *D. reticulatus*. This suggests that *I. ricinus* activity in forests is primarily temperature-driven, whereas in meadows, other environmental factors, such as humidity, may play a more significant role in regulating its activity (Figure 2 and Figure 3). These differences likely reflect species-specific physiological adaptations. *I. ricinus* is generally more sensitive to high temperatures and prefers moderate temperatures and humid environments, where the risk of desiccation is minimized [29]. However, some studies suggest that this species possesses traits that facilitate adaptation to changing climatic conditions [30].

In addition, depending on the sex, ticks of the same species often exhibit different sensitivities to abiotic variables due to their distinct life-history strategies, which we also observed during our study (Figure 2). Consequently, females are more sensitive to desiccation risk and will adjust their activity thresholds, questing only when relative humidity and temperature are within a range that maximizes successful feeding and minimizes water loss [1,11,21].

Our study confirms that *I. ricinus* and *D. reticulatus* exhibit distinct seasonal activity patterns. *D. reticulatus* follows a bimodal activity cycle, with peaks in early spring (March–April) and autumn (September–November), with the latter being more pronounced. In contrast, *I. ricinus* reaches peak activity in spring, with the highest abundance recorded in April and May (Figure 2). These seasonal trends align with previous studies and are likely influenced by species-specific life cycles and host availability throughout the year [31,32,33]. The spring and autumn peaks in adult *I. ricinus* and *D. reticulatus* questing correspond closely to periods of high host activity. For both *I. ricinus* and *D. reticulatus,* adult peaks coincide with the seasonal movements and foraging of roe deer (*Capreolus capreolus*) and other cervids, which serve as principal hosts for adult-stage ticks. Similarly, the autumn surge of *D. reticulatus* aligns with the peak abundance of large and medium-sized mammals [23].

Notably, during our long-term monitoring of *D. reticulatus* populations in the Polesie National Park, we observed shifts in the dominance of seasonal peaks, suggesting potential interannual variability in activity patterns [23]. These findings, along with previous research, highlight the need for ongoing surveillance of *I. ricinus* and *D. reticulatus* populations to better understand the environmental and biological factors driving their seasonal dynamics.

## 5. Conclusions

Our findings highlight species-specific differences in environmental tolerance, seasonal activity rhythms, and habitat selection. We found that adult *I. ricinus* ticks exhibited a strong preference for forested habitats, particularly during spring. In contrast, *D. reticulatus* demonstrated broader thermal tolerance, showing a distinct bimodal seasonal pattern, with activity peaks in early spring and autumn, whereas *I. ricinus* showed a unimodal pattern with a single peak in spring.

Environmental factors significantly influenced tick abundance and activity. Temperature and humidity played crucial roles in shaping seasonal dynamics. Habitat type strongly affected species distribution, with *D. reticulatus* being more prevalent in open meadow environments and *I. ricinus* favoring the more humid and shaded conditions of the forest.

Also, our findings have important implications for public health and veterinary strategies, such as the targeted timing of acaricide applications, and may be useful for modeling tick-borne diseases. Future research should quantify host abundance and behavior in these habitats and conduct manipulative microclimate experiments to determine the precise physiological thresholds for tick activity.

## Data Availability

All data generated in this study is included within the manuscript.
